# Chemosensory genes identified in the antennal transcriptome of the blowfly *Calliphora stygia*

**DOI:** 10.1186/s12864-015-1466-8

**Published:** 2015-03-31

**Authors:** Olivia Leitch, Alexie Papanicolaou, Chris Lennard, K Paul Kirkbride, Alisha Anderson

**Affiliations:** National Centre for Forensic Studies, University of Canberra, Canberra, Australia; CSIRO Division of Ecosystem Sciences and Food Futures Flagship, Canberra, Australia; CSIRO Land and Water Flagship, Canberra, Australia; School of Chemical and Physical Sciences, Flinders University, Bedford Park, Australia; Current Address: Hawkesbury Institute for the Environment, University of Western Sydney, Richmond, Australia; Current Address: School of Science and Health, University of Western Sydney, Penrith, Australia

**Keywords:** *Calliphora stygia*, Blowfly olfaction, Transcriptome, Chemosensory proteins, Odorant receptors

## Abstract

**Background:**

Blowflies have relevance in areas of forensic science, agriculture, and medicine, primarily due to the ability of their larvae to develop on flesh. While it is widely accepted that blowflies rely heavily on olfaction for identifying and locating hosts, there is limited research regarding the underlying molecular mechanisms. Using next generation sequencing (Illumina), this research examined the antennal transcriptome of *Calliphora stygia* (Fabricius) (Diptera: Calliphoridae) to identify members of the major chemosensory gene families necessary for olfaction.

**Results:**

Representative proteins from all chemosensory gene families essential in insect olfaction were identified in the antennae of the blowfly *C. stygia*, including 50 odorant receptors, 22 ionotropic receptors, 21 gustatory receptors, 28 odorant binding proteins, 4 chemosensory proteins, and 3 sensory neuron membrane proteins. A total of 97 candidate cytochrome P450s and 39 esterases, some of which may act as odorant degrading enzymes, were also identified. Importantly, co-receptors necessary for the proper function of ligand-binding receptors were identified. Putative orthologues for the conserved antennal ionotropic receptors and candidate gustatory receptors for carbon dioxide detection were also amongst the identified proteins.

**Conclusions:**

This research provides a comprehensive novel resource that will be fundamental for future studies regarding blowfly olfaction. Such information presents potential benefits to the forensic, pest control, and medical areas, and could assist in the understanding of insecticide resistance and targeted control through cross-species comparisons.

**Electronic supplementary material:**

The online version of this article (doi:10.1186/s12864-015-1466-8) contains supplementary material, which is available to authorized users.

## Background

In insects, volatile chemical cues (odours) provide information about food, reproduction [1], host selection, oviposition (egg/larvae laying) [2,3], and toxic compound avoidance [4]. As with other insects, olfactory-mediated behaviours are central to the ecology of blowflies [5-7]. Characterised by their larvae’s ability to develop on flesh, blowflies have significant roles in forensic entomology [8], agriculture [9-11], and medicine [12,13], and rely heavily on the detection of mammalian decomposition odours. The decomposition process produces a diverse array of inorganic gases and volatile organic compounds (VOCs). The VOCs produced cover almost all chemical families, including cyclic and non-cyclic hydrocarbons, oxygenated compounds (alcohols, ketones, aldehydes), nitrogen and sulfur containing compounds, acids/esters, halogens, and ethers [14-20]. It is believed that blowflies are capable of detecting decomposition odour immediately after death [21]. While it is acknowledged and accepted that chemical cues play a significant role in mediating many aspects of blowfly behaviour, the specific odours responsible are still to be elucidated.

Behavioural and physiological studies have shown the olfactory attraction of blowflies to whole host samples (e.g. liver, mice, pigs, etc.) [5,22] as well as their ability to detect (“smell”) individual odour molecules emitted from those samples [23-26]. Of the numerous VOCs available for detection, sulfur compounds appear to be some of the most important. Sulfides are consistently identified within the odour space of mammalian decomposition [14,17,19,27-29], are produced by plants that mimic decomposition odour [30], and have been shown to elicit physiological and behavioural responses in a variety of blowfly species [16,23,31-33]. Knowledge regarding the underlying molecular mechanisms regulating blowfly olfaction, however, is severely limited.

**Figure 1:**
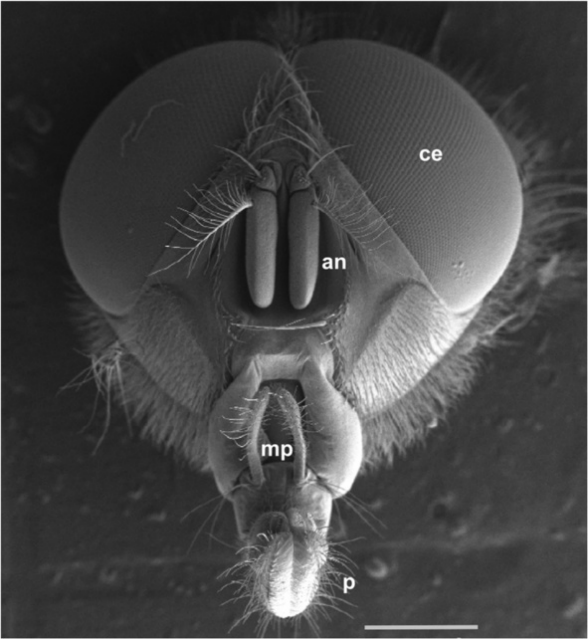
**Scanning electron micrograph of the head of a male** ***C. stygia*** **.** The main olfactory organs, the antennae (an) and maxillary palps (mp), are located between the compound eyes (ce) and the base of the proboscis (p), respectively. Scale bar: 1 mm.

Insects, including blowflies, sense odours via olfactory receptor neurons (ORNs) that are housed within chemosensory sensilla located primarily on the antennae and, to a lesser extent, the maxillary palps (Figure 1) [34]. Olfactory signal transduction – where environmental chemical signals are converted into electrical signals interpreted by the nervous system – starts with the binding of odour molecules by receptor proteins bound to ORN dendrites. There are three antennal receptor protein gene families that bind odour molecules, namely odorant receptors (ORs) [35], gustatory receptors (GRs) [36,37], and ionotropic receptors (IRs) [38]. Several non-receptor protein families have also been identified to be involved in invertebrate olfaction, including: sensory neuron membrane proteins (SNMPs) [39,40]; odorant binding proteins (OBPs) [41,42]; chemosensory proteins (CSPs) [43,44]; and odorant degrading enzymes (ODEs) [45]. Extensive information regarding the genes involved in olfaction, including their characteristics and potential roles, are provided by [37-39,44,46,47].

Because of the sequence diversity of olfactory genes, their identification has largely been only possible with insects for which genomic data is available [48,49]. However, recent advances in RNA-Seq and computational technologies have opened up such identifications in non-model organisms. This has resulted in the identification of olfactory genes in a wide range of insects for which no sequenced genome is available [50-56]. With respect to blowflies, very few chemosensory genes have been identified [57,58]. For example, the co-receptor crucial for the appropriate function of ligand-binding ORs has been identified in various blowfly species [59-61] along with two candidate ligand-binding ORs [62]. Candidate genes from the OBP, IR, and GR families have also been identified [59,63]. However, no research regarding the functional characterisation of individual olfactory genes has been published and the ligands for these candidate genes remain unidentified.

This research investigated the antennal chemosensory gene families of the blowfly *Calliphora stygia* (Fabricius) (Diptera: Calliphoridae) via transcriptomic analysis. *C. stygia* is native to Australia and is primarily carrion-dependent with respect to its feeding and reproductive behaviour [64]. Previous research with *C. stygia* has focused primarily on factors affecting its growth throughout its lifecycle [65-68]; there is no information regarding its olfactory abilities. Identification of members of the primary gene families mediating insect olfaction permits a better understanding of the molecular basis of blowfly olfaction. Such knowledge could ultimately lead to the identification of new targets of control strategies [11,57], an improved understanding of how blowflies recognise, locate, and colonise hosts, as well as improved methods for estimating post-mortem interval [7].

## Results

### Antennal transcriptome

**Figure 2:**
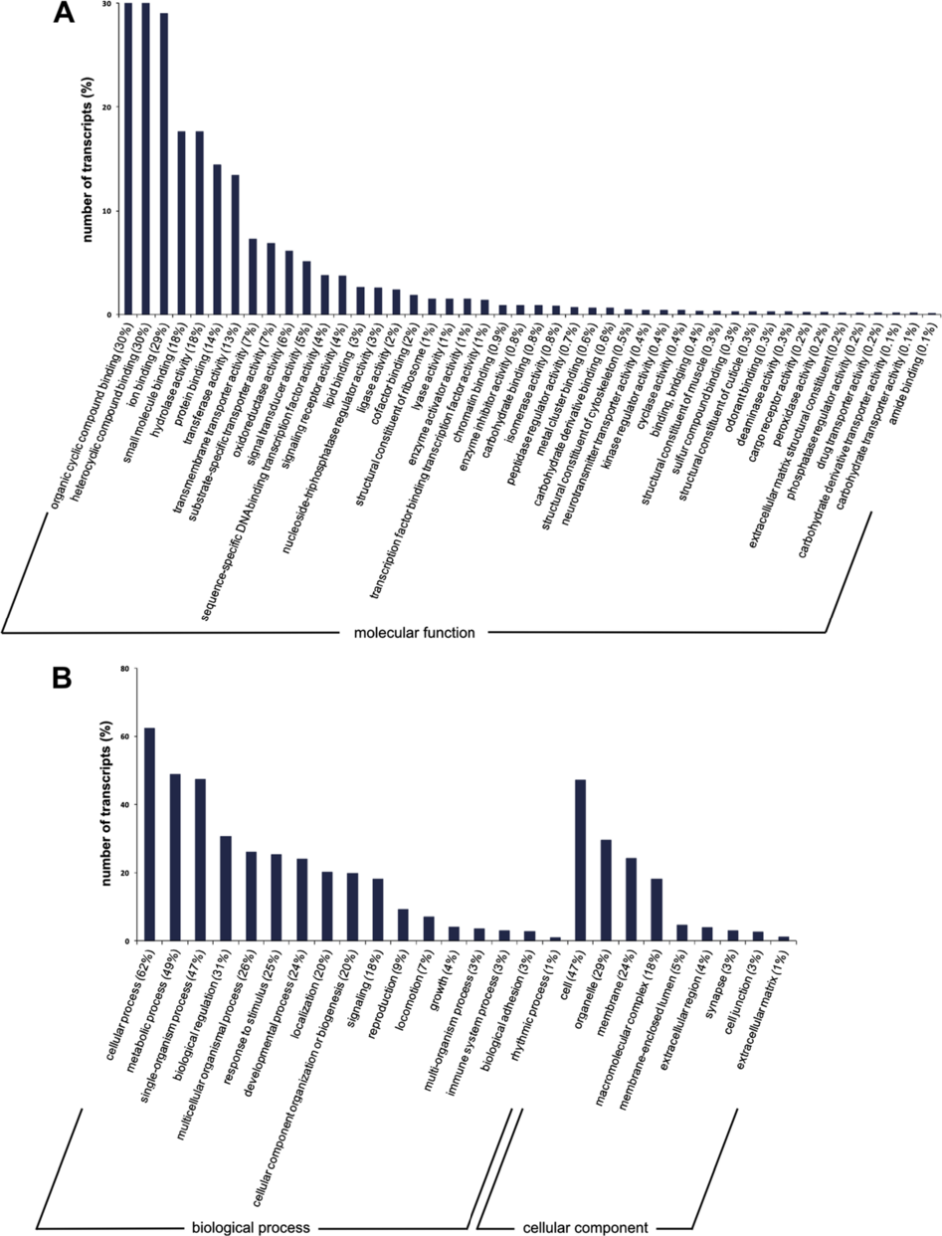
**Distribution of** ***C. stygia*** **antennal transcriptome data in GO terms.** GO analysis of 8,709 (8,628 male, 8,609 female) transcripts for their predicted involvement in molecular functions **(A)** and biological processes **(B)** or as cellular components **(B)**. GO categorisation for molecular functions is presented at level 3 and at level 2 for biological processes and cellular components. Annotated genes are depicted as percentages of the total number of transcripts with GO term assignments.

The combined Trinity assembly of the male and female *C. stygia* antennal transcriptomes led to the generation of 75,836 contigs, from which 16,522 non-redundant putative transcripts were predicted. Searches against the NCBI non-redundant protein database returned 14,094 transcripts showing sequence similarity to known proteins (Additional file 1). Of these, 8,709 (~53% of all predicted proteins) were assigned at least one GO term (Figure 2). There was no significant difference between the male and female data sets with respect to GO annotation therefore the male and female data sets are presented together. The most abundant GO term associations were in relation to basic cell functions; however, GO terms associated with olfaction (e.g. “odorant binding”, “response to stimulus”, and “signal transducer activity”) and enzyme activity (e.g. “hydrolase activity”, “transferase activity”, etc.) were also represented within the data sets. The large number of transcripts without associated GO terms (7,813 transcripts, ~47%) potentially represent orphan genes.

### Identification of candidate odorant receptors

Analysis of the *C. stygia* antennal transcriptomes identified 48 and 50 candidate OR proteins in the male and female data sets respectively (combined total of 50 candidates [GenBank accession numbers KJ702047-KJ702096], with CstyOR118 and CstyOR119 being absent from the male data set). Additional file 2: Table S1 summarises transcript name, length, best BLASTx hit, predicted domains, and male or female specificity. Twenty-four of the putative CstyORs likely represent full-length sequences. The majority of partial length transcripts possess overlapping regions with low amino acid sequence identity, which indicates that they represent separate individual proteins. However, the possibility that the remaining non-overlapping transcripts represent fragments of individual proteins cannot be excluded; therefore, based on sequence alignments and subsequent fragment location (i.e. C-terminus, internal, or N-terminus), the total number of CstyORs reported could be reduced by two.

Consistent with the diversity of the OR gene family (with the exception of Orco), full length putative *C. stygia* ORs shared between 9% and 49% amino acid identity (average 16%). Predictive software also indicated full-length candidate CstyOR transcripts possess between three and eight transmembrane domains. Depending on the length of the partial transcripts, the remaining CstyORs were predicted to have zero to seven transmembrane domains (Additional file 2: Table S1).

**Figure 3:**
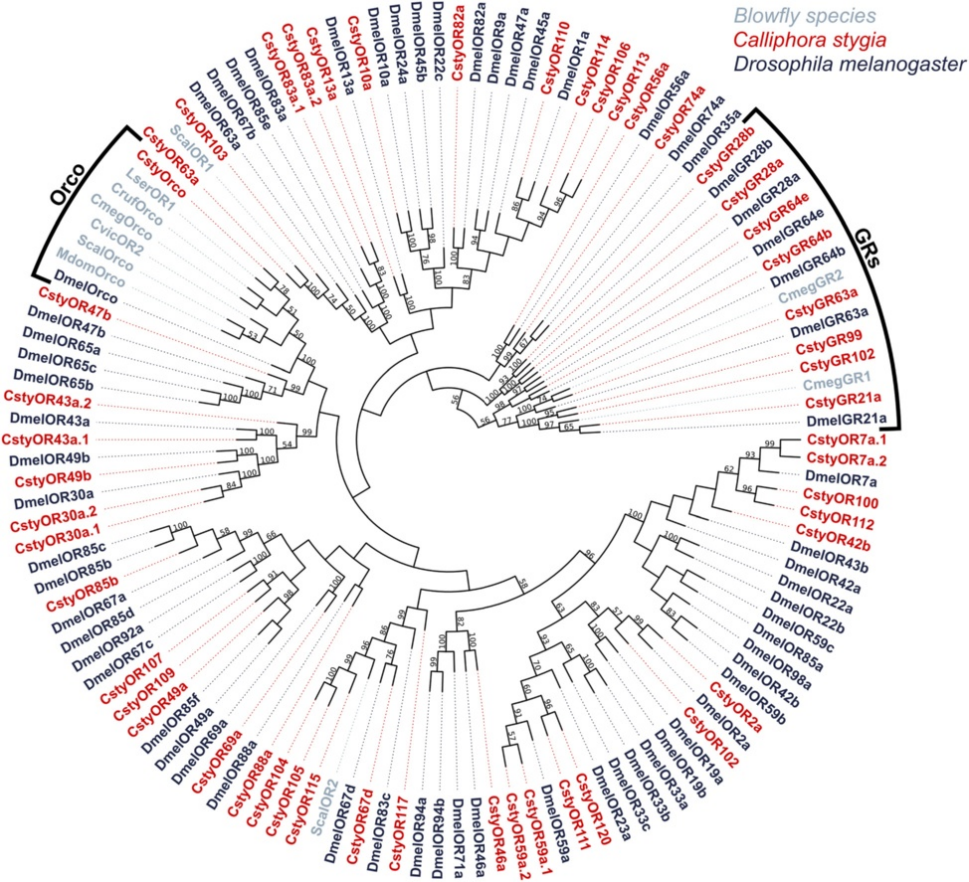
**Odorant and gustatory receptors.** Maximum-likelihood tree of candidate *C. stygia* ORs and GRs (red) with ORs and GRs (putative GR orthologues only) identified in *Drosophila melanogaster* (dark blue) and various blowfly species, including *Musca domestica* (Mdom), *Calliphora vicina* (Cvic), *Chrysomya megacephala* (Cmeg), *Chrysomya rufifacies* (Cruf), *Lucilia sericata* (Lser), and *Stomoxys calcitrans* (Scal) (grey). Distinct Orco and GR clusters are apparent.

Importantly, the highly conserved co-receptor Orco was identified in the *C. stygia* transcriptomes, sharing ~88% to ~99% amino acid sequence identity with Orco’s from *Drosophila melanogaster* and other blowfly species. As expected, greater sequence identity was observed with other blowfly Orco’s than with *Drosophila* (Figure 3). Putative *D. melanogaster* orthologues could be assigned for the majority of the presumably ligand-binding CstyORs; however, 13 appear to have no *D. melanogaster* counterpart. Of these 13, ten could be assigned putative orthologues (based on reciprocal best hits) in other species, including *Anopheles gambiae Bombyx mori*, *Danaus plexippus*, and other *Drosophila* species. Interestingly, a putative orthologue of DmelOR67d, a pheromone specific receptor, was identified in *C. stygia* (sharing ~43% amino acid sequence identity).

In absolute terms (i.e. presence or absence), there were no significant differences in the number of candidate OR proteins identified in the respective male and female data sets. Quantitative differences in the relative transcript abundances were observed (Additional file 3). Of the identified candidate OR transcripts, nine appear to be enriched (i.e. double the normalised FPKM value) in the female data set, while 15 are enriched in the male data set.

### Identification of candidate gustatory receptors

Twenty-one candidate GR transcripts were identified in the combined male and female *C. stygia* transcriptomes (Additional file 2: Table S2) (a total of 20 male and 20 female candidates [GenBank accession numbers KJ702097-KJ702117]). The majority of candidate CstyGRs were partial fragments (only five represent full-length proteins), encoding overlapping but distinct sequences. This establishes the proteins as being fragments of independent genes. Consistent with other insect GRs [69], transmembrane domain and topology predictions in full-length transcripts indicated between six and eight domains with an intracellular N-terminus and extracellular C-terminus being the most likely configuration. The eight candidate CstyGR transcripts included in the phylogenetic analysis formed a distinct clade (Figure 3); none clustered within the OR clades thus indicating that the transcripts are more related to GRs than ORs. The CstyGRs were also observed to group with their presumed *Drosophila* orthologues, which have been shown to have roles in carbon dioxide detection (GR21a and GR63a) [36,70] and thermosensation (GR28b) [71], or are members of the candidate sugar GR64 receptor subfamily (GR64b and GR64e) [72]. Several of the partial length candidate CstyGRs also show high sequence amino acid similarity to known sugar (DmelGR43a) and bitter (DmelGR66a and DmelGR93a) *Drosophila* receptors (Additional file 2: Table S2).

### Identification of candidate ionotropic receptors

**Figure 4:**
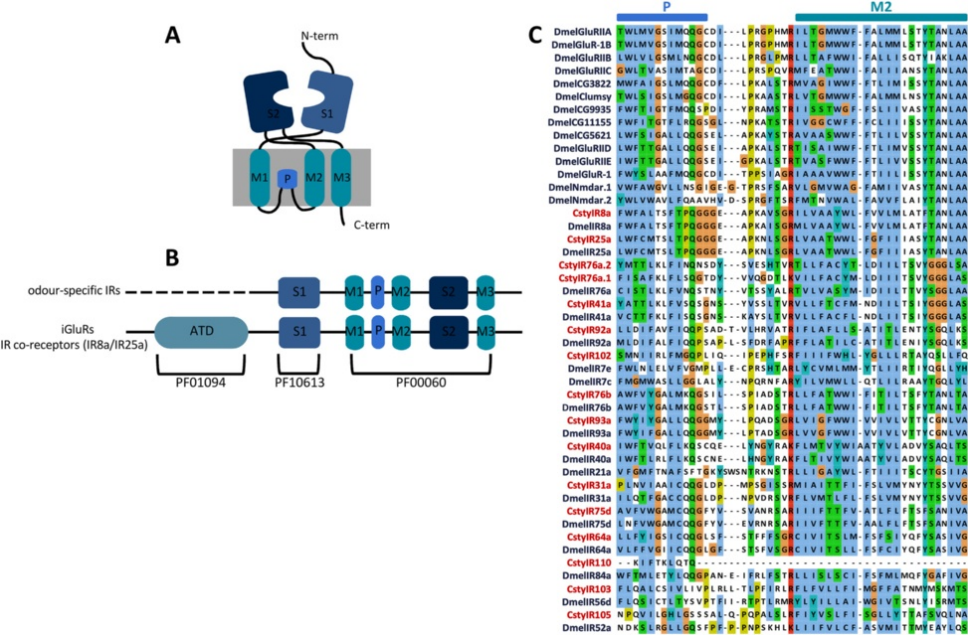
**Ionotropic receptors.** Predicted protein domain organisation of IRs and iGluRs/IR co-receptors illustrated in diagram **(A)** and linear **(B)** form with associated Pfam predicted domains (adapted from [23,54]). **(C)** MAFFT amino acid alignment of the ion channel pore (P) and second transmembrane (M2) domains of candidate *C. stygia* IRs (red) and *Drosophila melanogaster* IRs and iGluRs (blue).

**Figure 5:**
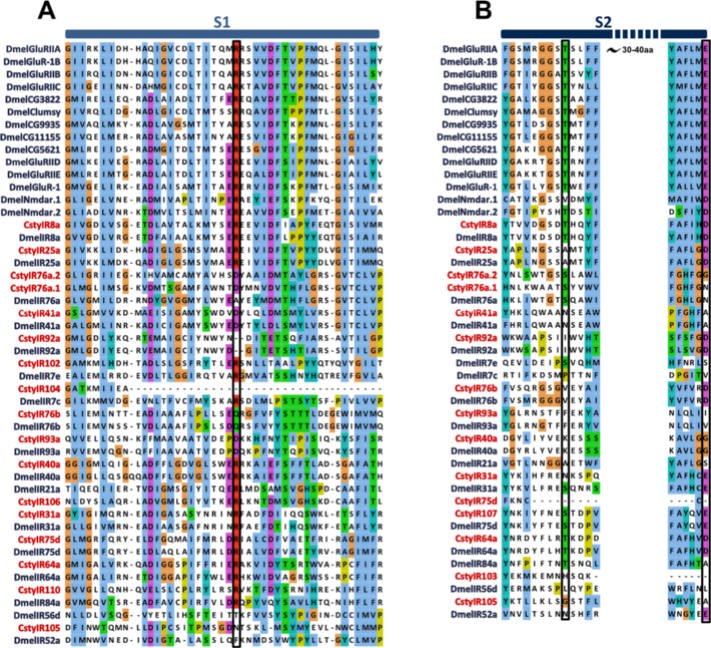
**Ligand-binding S1 and S2 domains.** Important glutamate-interacting residues are lacking in the ligand binding domains of most *C. stygia* IR candidates. MAFFT amino acid alignments of the S1 **(A)** and part of S2 **(B)** ligand binding domains of candidate *C. stygia* IRs and *Drosophila melanogaster* IRs and iGluRs. The key binding residues in iGluRs are boxed.

Twenty-two candidate IRs were identified in both the male and female *C. stygia* antennal transcriptomes (Additional file 2: Table S3 [GenBank accession numbers KJ702118-KJ702139]). Structural analysis and amino acid sequence alignments revealed that most candidate CstyIRs shared the structural organisation of insect IRs and iGluRs (in the case of co-receptors IR8a and IR25a) (Figure 4A and B). The most conserved sequence regions were the three transmembrane domains and the ion channel pore (Figure 3C) [38,73]. Characteristic variability of the glutamate-binding residues located in the ligand-binding S1 and S2 domains was also present (Figure 5A and B). Only two CstyIRs (CstyIR8a and CstyIR64a) retain all residues characteristic of iGluRs (R, T and D/E) [38]; all other IRs have a diversity of amino acids at one or more of these positions indicating variable ligand binding properties. However, it should be noted that some of the putative CstyIRs with incomplete sequences could not be assessed for the presence of these crucial residues.

**Figure 6:**
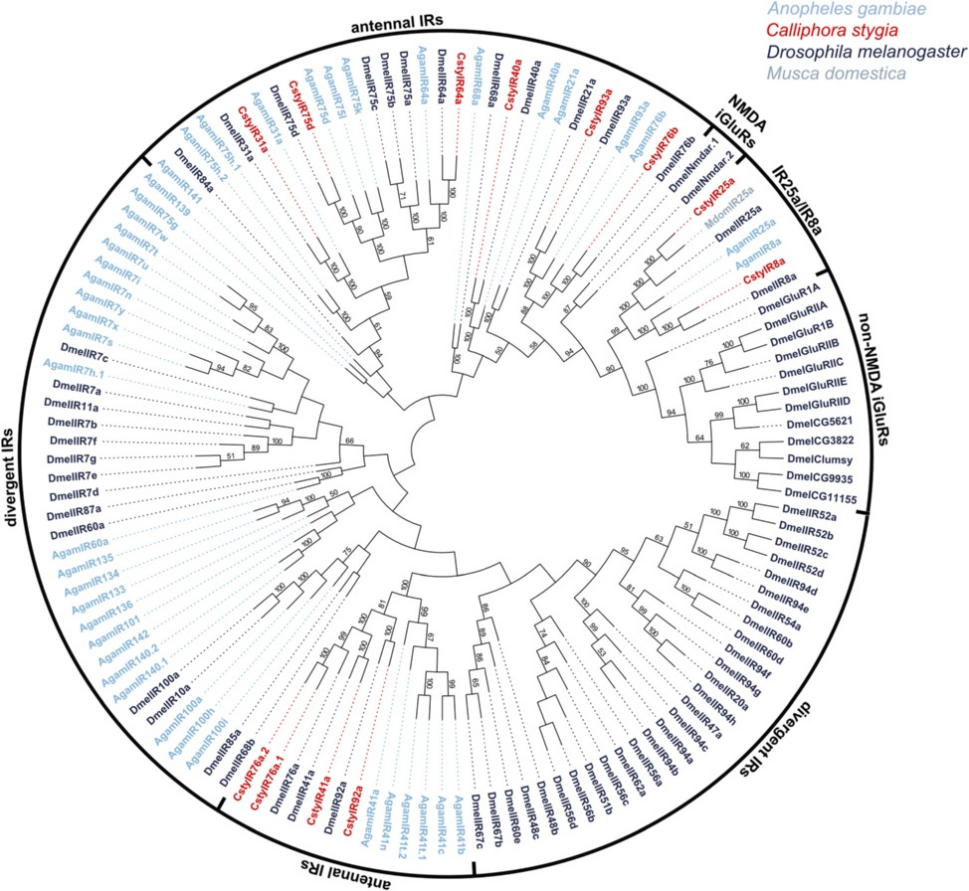
**Phylogenetic tree of a selection of Dipteran IRs.** Neighbour-joining tree of candidate *C. stygia* IRs (red) with IRs from *Anopheles gambiae* (light blue), all iGluRs and IRs from *Drosophila melanogaster* (dark blue) and the single IR identified in *Musca domestica* (grey). The identified IR candidates of *C. stygia* cluster with their presumed orthologues within the “antennal IR” clades and the IR25a/8a subgroup.

Phylogenetic analysis revealed that the candidate CstyIRs were more closely related to IRs then iGluRs, with all candidate CstyIRs assessed clustering with their presumed “antennal” orthologues (Figure 6). This analysis identified representatives from 10 of the 13 orthologous “antennal” IR groups conserved across the protostome species analysed by Croset *et al*. [73]. Thus, orthologues of the remaining three conserved groups (IR21a, IR60a, and IR68a) are either lacking from the *C. stygia* transcriptome assembly (due to their low expression levels [38,74] which could result in them being missed during random sequencing) or are yet to be identified within the putative CstyIRs represented by partial sequences (e.g. CstyIR101 and CstyIR106 share 78% and 70% identity with DmelIR21a, respectively). Notably, transcripts putatively encoding IR8a, IR25a, and IR76b – which are thought to function as IR co-receptors [38,75] – were found in *C. stygia* antennae. No candidate CstyIRs clustered within the “divergent” clade.

### Identification of candidate sensory neuron membrane proteins

**Figure 7:**
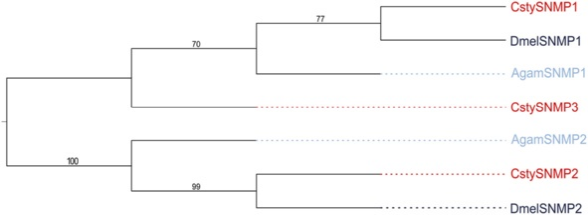
**Phylogenetic relationships of SNMPs.** Maximum-likelihood tree of candidate *C. stygia* SNMPs (red) with SNMPs from *Drosophila melanogaster* (dark blue) and *Anopheles gambiae* (light blue).

Analysis of the male and female *C. stygia* antennal transcriptomes identified three candidate SNMPs present in both data sets (Additional file 2: Table S4 [GenBank accession numbers KJ702172-KJ702174]), two of which (CstySNMP1 and CstySNMP3) likely represent full-length genes. Notably, a putative orthologue to the *D. melanogaster* protein, SNMP1, which has been shown to have an important role in pheromone detection [39], was present in the *C. stygia* data sets (Figure 7).

### Identification of putative odorant binding proteins

**Figure 8:**
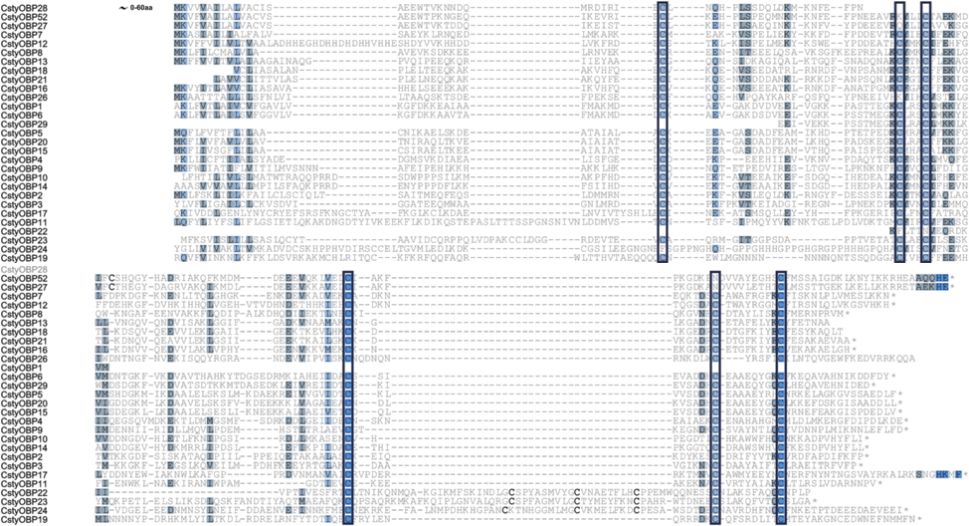
**Candidate** ***C. stygia*** **OBPs exhibit all three motif sub-types.** The conserved six cysteines present in the classic motif are boxed. The potential alternative conserved cysteines in the Minus-C sub-group and the additional cysteines present in the Plus-C motif are in bold text.

Twenty-eight candidate OBP transcripts were identified in *C. stygia* (Additional file 2: Table S5), all of which were present in both the male and female data sets [GenBank accession numbers KJ702140-KJ702167]. Of the 18 full-length CstyOBPs, 15 exhibited the classic arrangement of conserved six-cysteines, 1 was the Plus-C gene motif (CstyOBP23), and 2 were Minus-C (CstyOBP25 and CstyOBP27) (Figure 8) [76,77]. A further 6 Classical, 2 Plus-C (CstyOBP22 and CstyOBP24), and 1 Minus-C (CstyOBP26) type transcripts could be allocated from the partial CstyOBP transcripts. Notably, while the CstyOBPs classified as Minus-C do lack one or more of the cysteine residues in the conserved classic locations, additional cysteines (conserved in the CstyOBP Minus-C motif sequences) were present in nearby positions that may act as an alternative.

**Figure 9:**
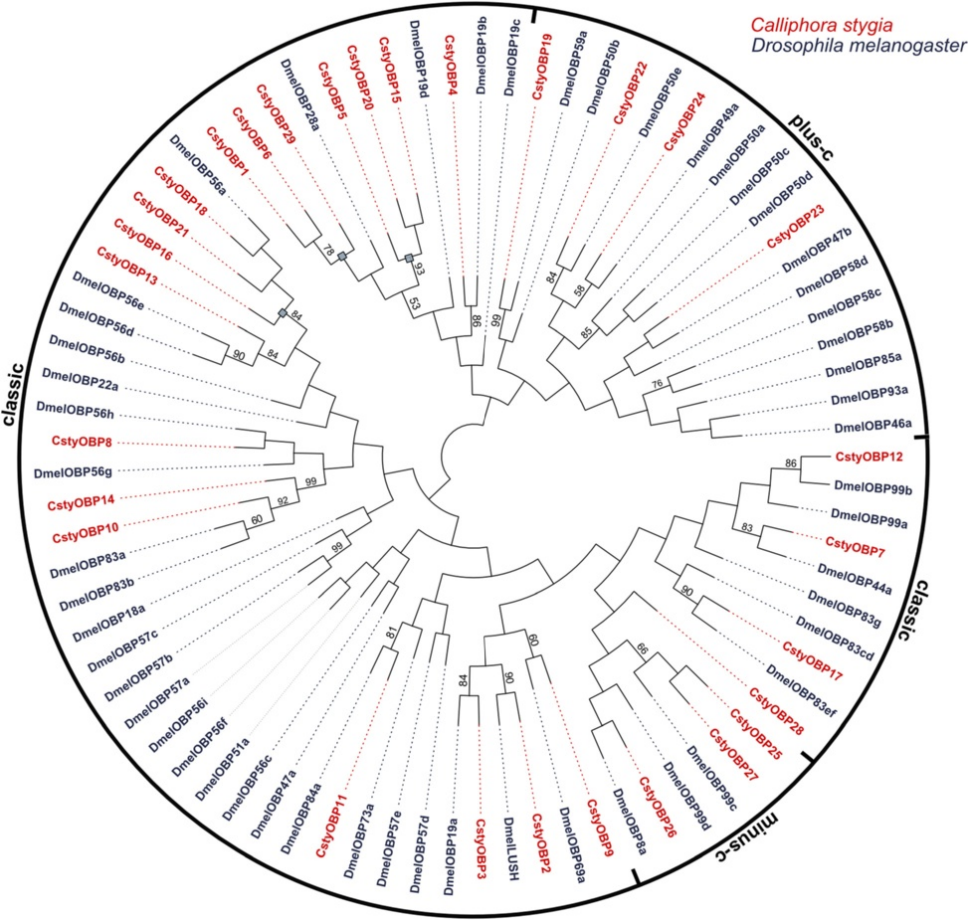
**Odorant binding proteins.** Phylogenetic tree of candidate CstyOBPs (red) with *Drosophila melanogaster* OBPs (dark blue). CstyOBPs cluster within their respective OBP sub-families with classic CstyOBPs spread throughout various clades. Squares indicate the putative blowfly specific clades related to DmelOBP22a and DmelOBP19b-d.

Phylogenetic analysis revealed that all candidate CstyOBPs clustered in accordance with their respective sub-families (Figure 9). This analysis also indicated that *D. melanogaster* orthologues were likely to be present for many of the putative CstyOBPs, although a few small *C. stygia* specific clades potentially indicate a level of divergence within the blowfly. The low average amino acid identity exhibited by the candidate CstyOBPs is consistent with that of other species [76,78] and aligns with the notion that their role involves interacting with a range of diverse odour molecules. Interestingly, one particular candidate CstyOBP shared significant amino acid sequence identity to the *D. melanogaster* OBP LUSH (DmelOBP76a) (55% identity), which, in addition to its role in driving the avoidance of high alcohol concentrations [79], has been shown to play a role in pheromone sensitivity [39,80].

### Identification of candidate chemosensory proteins

**Figure 10:**
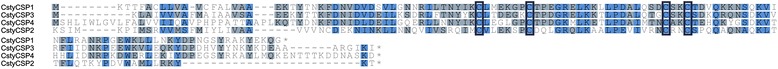
**Alignment of candidate** ***C. stygia*** **CSPs.** All candidate *C. stygia* CSPs exhibit the highly conserved four-cysteine profile. The four conserved cysteine residues are boxed.

Four transcripts encoding candidate CSPs were identified in both the male and female *C. stygia* transcriptomes (Additional file 2: Table S6), three of which likely represent full-length proteins [GenBank accession numbers KJ702168-KJ702171]. All of the identified amino acid sequences possessed a signal peptide and the highly conserved four-cysteine profile (Figure 10).

### Identification of candidate odorant degrading enzymes

GO annotation of the transcriptomes indicated an enrichment of proteins involved in catalytic activity. Further analysis of the combined male and female transcripts identified 136 candidate Cytochrome P450s and esterases (Additional file 2: Table S7 [GenBank accession numbers KJ702175-KJ702310]). Of the 39 candidate esterases (37 of which were annotated from both the male and female data sets), 17 likely represent full-length sequences. Ninety-three of the 97 candidate P450s were present in both sexes with 28 sequences predicted to be full-length.

**Figure 11:**
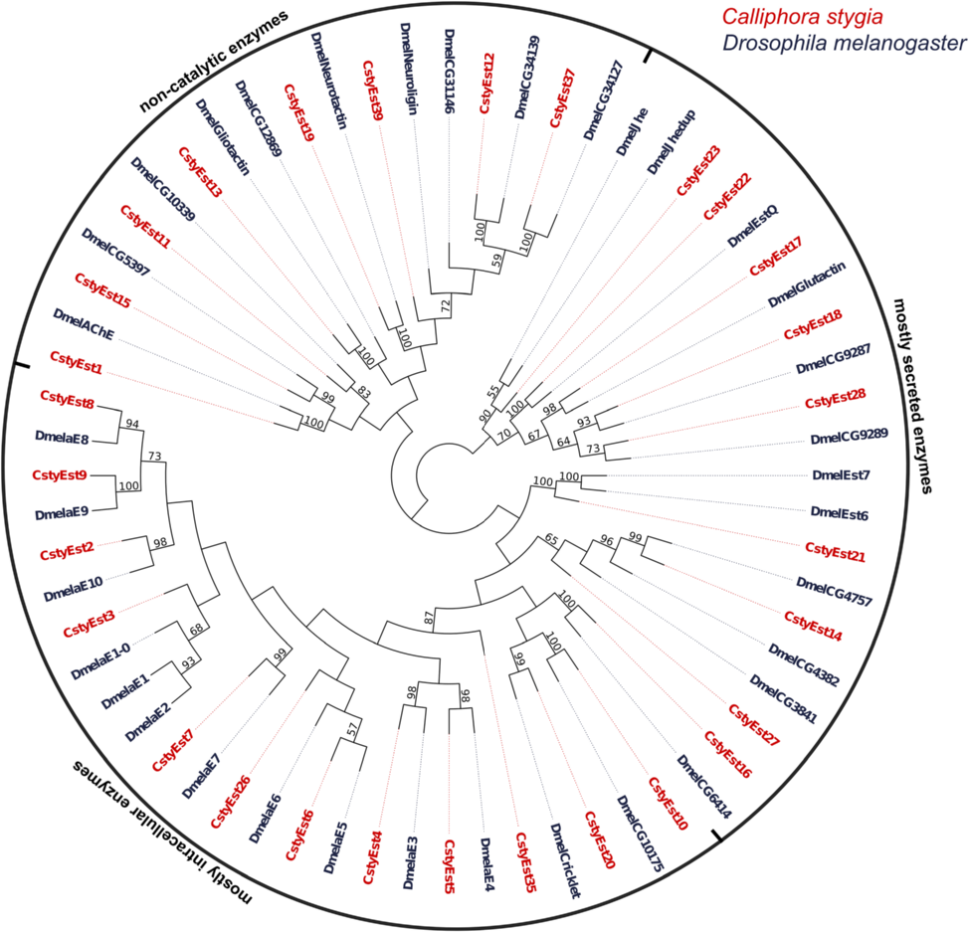
**Phylogenetic tree of esterases.** Maximum-likelihood tree of candidate *C. stygia* esterases (red) with esterases from *Drosophila melanogaster* (dark blue). The candidate esterases identified in *C. stygia* are spread throughout all three of the major esterase gene family groups [47], indicating a variety of possible antennal functions.

**Figure 12:**
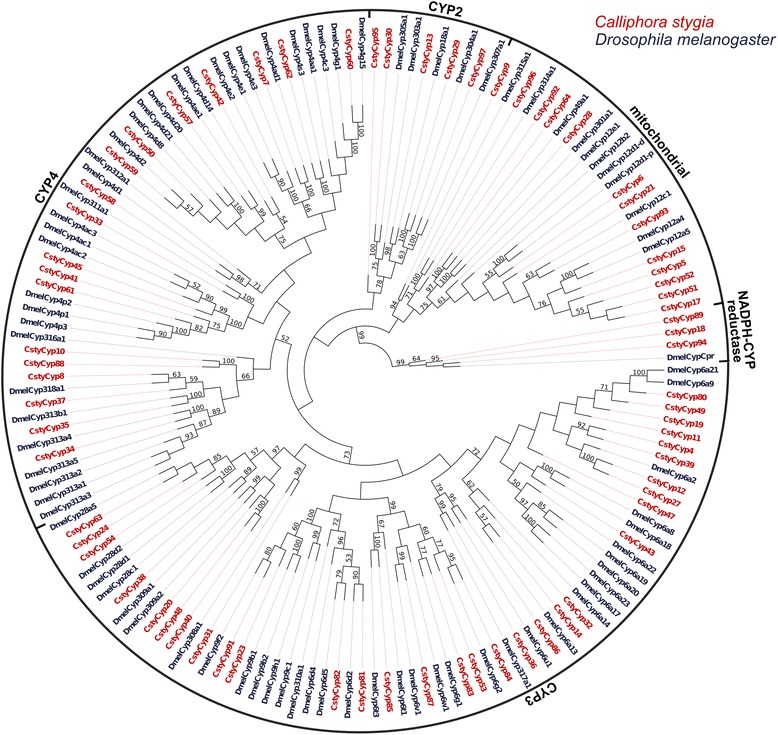
**Cytochrome P450s.** Maximum-likelihood tree of candidate *C. stygia* Cytochrome P450s (red) with *Drosophila melanogaster* Cytochrome P450s (dark blue). The identified CypP450 candidates from *C. stygia* cluster within the four distinct clades (CYP2, CYP3, CYP4, and mitochondrial). Candidate NADH-Cytochrome reductases were also identified.

Phylogenetic analysis revealed that the candidate CstyEsts clustered within all three of the major functional groups of the esterase gene family (based on the classification system of [47]) (Figure 11). This indicates that the CstyEsts have possible functions in neurodevelopment (non-catalytic enzyme group), detoxification (mostly intracellular enzyme group), and hormone and pheromone processing (mostly secreted enzyme group) [47]. This analysis also indicated that *D. melanogaster* orthologues were likely to be present for many of the candidate CstyEsts, with no apparent *C. stygia* specific clades or expansions. Candidate CstyCyps were also distributed throughout the four major Cytochrome P450 gene family groups, specifically the CYP2, CYP3, CYP4, and mitochondrial clades (as classified by [81]) (Figure 12). Genes from the CYP3 clade, in which the majority of CstyCyps clustered (31 transcripts), have been shown to be involved in xenobiotic metabolism and insecticide resistance [82]. Additionally, some genes from the CYP4 clade have been associated with the metabolism of odorants or pheromones [82]. Candidate NADPH-Cytochrome reductases, proteins required for the reduction of P450s, were also identified. Notably, several of the candidate enzymes shared significant amino acid sequence identity to CYP450s and esterases specifically associated with pesticide resistance and detoxification. For example, the candidate esterase CstyEst7 was a best BLASTx hit to *Lucilia cuprina’s* E3 (sharing 89% amino acid identity), an enzyme shown to be a prime candidate for pesticide resistance [83,84]. Additionally, CstyCyp83 was a reciprocal best hit to *D. melanogaster’s* CYP6g1, which has been associated with DDT resistance [85]. While it is possible that many of the candidate enzyme transcripts are ODEs, significant biochemical analysis is necessary to identify their specific physiological roles.

## Discussion

This research represents the first comprehensive analysis of a blowfly antennal transcriptome for the purpose of identifying members of the major chemosensory gene families necessary for olfaction. The reported gene sets therefore represent a significant addition to the data regarding the molecular basis of blowfly olfaction. Due to the importance of olfaction in blowfly behaviour, the identified genes could represent novel targets for future population control methods as well as providing opportunities for improving post-mortem interval estimations through a greater understanding of the odour related factors that favour or inhibit blowfly detection and colonisation.

Classification of predicted functions of the male and female *C. stygia* transcripts via GO assignment produced similar results as those obtained for other invertebrates [50,52,62,86,87]. The number of individual candidate transcripts identified for many of the olfactory gene families is also comparable to those of other Dipteran, Coleopteran, and Lepidopteran species for which the antennal transcriptome has been examined [50,51,86,88-91]. Without considering the potential significance of individual genes to each species studied, the similarity of the different data sets does indicate a certain level of antennal conservation with respect to gene expression.

Interestingly, the 50 candidate ORs identified in *C. stygia* show greater similarity to the numbers identified in Lepitopteran species (43 in *Cydia pommonella*, and 47 in *Manduca sexa*, *H. armigera*, and *Spodoptera littoralis*) [51,86,88,92] than to the closer related *D. melanogaster* (37 ORs) [91]. Additionally, the number of candidate *C. stygia* GRs (20) and IRs (22) is greater than those reported for most species [52,91-93]. While these studies also analysed antennal transcriptomes, variations in the number of transcripts identified could arise from differences in the sequencing methods, sequencing depth, and/or sample preparation. The greater number of candidate ligand-binding transcripts annotated in *C. stygia* could be due to ecological differences; however, further research is required to determine the specific reason such differences may exist. Typically, invertebrates with large numbers of antennal GRs use their antennae for tasting purposes as well as for olfaction (e.g. the butterfly *Heliconius melpomen*e) [94]. However, there are no reports of blowflies exhibiting such behaviour. Interestingly, recent transcriptomic analysis of *A. gambiae* also identified GRs in addition to the previously identified carbon dioxide receptors [90]. While primarily linked to the detection of tastants [95], the *C. stygia* and *A. gambiae* transcriptomes, and the increasing range of “non-gustatory” sensory functions being identified for these proteins [96], suggests antennal GRs could have far more diverse roles.

Functional analysis of *D. melanogaster* IRs has demonstrated their role in the detection of amines and acids [38,97], which are significant compounds emitted during biological decomposition [14,19,28,98]. Candidate *C. stygia* IRs putatively orthologous to *D. melanogaster* IRs shown to respond to individual decomposition compounds (e.g. propanoic acid, ammonia, butyric acid, and putrescine) [93] were present amongst the male and female *C. stygia* data sets. Predicting the ligands to which olfactory receptors will respond based on empirical data from other receptors is problematic due to their extensive divergence. Therefore, determining receptor ligands can only be achieved experimentally. Ultimately, the reason for such a large number of ORs, GRs, and IRs in *C. stygia* is unknown and additional molecular biology and functional experiments are required in order to confirm the expression and role of these genes. Overall, the comparable and/or greater number of genes identified within each of the olfactory gene families suggests that a comprehensive antennal data set has been obtained for *C. stygia*. Such results also illustrate the sensitivity and value of transcriptomic analysis via next generation RNA-Seq for non-model organisms.

Notably, the *C. stygia* transcriptome data indicates that the chemosensory gene repertoire is largely similar in the male and female. This indicates that male and female *C. stygia* share similar odour-coding capacity. Quantitatively, the range of relative expression levels (i.e. low to high expression) of the candidate ligand-binding ORs in *C. stygia*, in relation to Orco, are similar to those reported for other Dipteran species [91,99,100]. However, preliminary data suggests that there is a difference in the relative levels of expression of individual ORs between male and female *C. stygia* (Additional file 3). Therefore, while male and female antennae likely perceive similar odour stimuli, their sensitivities, and hence the odour significance to the male and female, may differ. This is consistent with previous studies, which show that electrophysiological responses can be elicited from males and females by a particular odour [23,101], while leading to sex-based behavioural differences [22,102-104]. Additional biological repeats and experimental validation (e.g. quantitative PCR) are required to confirm the expression data. Further research, such as *in situ* hybridization and single-sensilla recordings, would also be beneficial to determine the distribution and frequency of ORs within the antennae.

Sexually dimorphic expression of chemosensory genes could indicate roles in sex-specific behaviours, including those mediated by pheromones. Invertebrate pheromone detection mediates various behaviours including aggregation, mate recognition, and sexual behaviour [105]. In *D. melanogaster*, reception of the male volatile pheromone, cis-vaccenyl acetate, is achieved with LUSH, OR67d, and SNMP1 [1,39,80]. The identification of putative orthologues of these three proteins in *C. stygia* could indicate potential functional pheromone detection in this species. Proteins sharing high sequence similarity to LUSH and DmelOR67d have also been identified in *Stomoxys calcitrans* (stable fly) [62]. Candidate blowfly pheromones have been described for several species [106-108]; however, all have been non-volatile cuticular hydrocarbons and therefore are more likely to be contact pheromones. Additionally, the mechanisms that allow pheromone reception in blowflies are not yet known. Further characterisation of the candidate *C. stygia* proteins is required to determine if they exhibit similar chemosensory roles to the *D. melanogaster* genes or if they have adapted different functions.

## Conclusions

A total of 264 transcripts encoding putative chemosensory proteins from the seven major olfactory gene families were annotated in the blowfly *C. stygia*. The transcriptomic approach proved to be a highly effective strategy for the identification of divergent blowfly chemosensory receptors for which no genomic data is publically available. Comparative analysis with other species suggests that near-complete information regarding the molecular basis of *C. stygia* olfaction was obtained. This research greatly improves the gene inventory for *C. stygia* and provides a valuable resource for future analysis on blowfly olfaction. Such information will be fundamental for future comparative analyses that could highlight interspecies differences underlying ecological differences and genetic adaption.

## Methods

### Insects

*Calliphora stygia* pupae were obtained from a commercial supplier (Sheldon’s Bait, Victor Harbor, SA, Australia) and maintained at 23°C with a 12 hr light: 12 hr dark photoperiod. Following eclosion, males and females were separated and provided with water and protein biscuits (sugar, eggs, powdered milk yeast, and water) as per published procedures [109].

### RNA extraction and reference transcriptome generation

Antennae were excised from adult male and female flies (minimum 5 days old) following snap freezing in liquid nitrogen. Total RNA was isolated using the RNAqueous®-Micro Kit (Ambion) with DNase treatment following the manufacturer’s protocol. RNA quantity was determined on a Nanodrop ND-2000 spectrophotometer (Thermo Scientific, Waltham, MA, USA). Synthesis of cDNA and Illumina library generation was completed at BGI – Hong Kong Co., Ltd. using Illumina HiSeq2000 sequencing. Raw RNA-Seq data was pre-processed, combined, and *de novo* assembled using Trinity [110,111]. Open reading frames were predicted using TranscriptDecoder software as implemented in Trinity. *In silico* expression profiles were generated using DEW [112], which aligns data using Bowtie2 v.2.1.0 [113] with RNA-eXpress post-processing [114]. Expression levels were expressed in terms of FPKM values (fragments per kilobase per million reads).

### Gene identification and annotation

An initial assessment of the combined male and female *C. stygia* transcriptomes was completed via searches against the NCBI non-redundant protein database (using BLASTp with a 1e^-10^ threshold) and GO Annotation with Blast2GO [115,116]. Putative chemosensory genes were identified by custom database nucleotide Blast profile searches (Geneious software) using known *D. melanogaster* sequences as queries. Putative *C. stygia* chemosensory genes were in turn used as queries to identify additional genes (tBLASTx and BLASTp). Iterative searches were completed until no new candidates were identified. Identification of candidate genes was verified by additional BLAST searches using the *C. stygia* contigs as queries against the NCBI non-redundant protein database (BLASTx). Protein domains (e.g. transmembrane domains, signal peptides, secondary structures, etc.) were predicted by queries against InterPro using the InterProScan Geneious software plugin running a batch analysis (e.g. HMMPanther, SignalPHMM, Gene3D, HMMPfam, TMHMM, HMMSmart, Superfamily, etc.) [117]. Membrane topology was assessed with Phobius [118]. Sequences were classified based on sequence similarity, domain structure predictions, and phylogenetic analysis.

### Orthology determination

*C. stygia* genes were defined as potential orthologues when they were reciprocal best hits with the corresponding *D. melanogaster* gene and subsequently grouped within the same clade in phylogenetic trees.

### Protein nomenclature

Candidate chemosensory transcript names are preceded by a four-letter species abbreviation in accordance with established conventions (e.g. [73,119]). *C. stygia* transcripts deemed orthologous (based on sequence similarity) to *D. melanogaster* sequences were given the same name (e.g. DmelIR8a, CstyIR8a, DmelOrco, CstyOrco). Multiple copies of a putative *D. melanogaster* orthologue were given the same name followed by a point and number (e.g. CstyIR76a.1, CstyIR76a.2). For IRs, novel transcripts (i.e. those without putative orthologues) were numbered from 101 upward in order to avoid confusion (*D. melanogaster* IRs are numbered up to IR100a). Similarly, novel ORs and GRs were numbered from 99 upwards. Transcripts identified as putative OBPs were named according to previously established conventions [80]. Briefly, OBPs were numbered from one upwards in the following order: “classical” members; “Plus-C” members; and “Minus-C” members. OBPs unable to be classified (due to incomplete sequences) were listed last. Candidate Cytochrome P450s and esterases were numbered from one upwards.

### Phylogenetic analysis

Amino acid sequences were aligned using MAFFT [120]. Unrooted neighbour-joining (for IRs) and maximum-likelihood trees (for ORs and GRs, OBPs, SNMPs, esterases, and Cytochrome P450s) were constructed using MEGA5 [121] and subsequently viewed and graphically edited in FigTree v1.4.0 [122] and InkScape v0.48.2 [123]. Branch support was assessed using the bootstrap method based on 1000 replicates. Incomplete transcripts without sufficient overlap in alignments and transcripts less than 200 amino acids in length (except for the OBPs where full-length transcripts are generally shorter than 200 amino acids) were excluded from phylogenetic analyses to ensure that the analysed transcripts corresponded to individual genes and that greater accuracy in the analyses was maintained.

Phylogenetic trees were based on Dipteran data sets. The IR data set contained 12 *C. stygia* sequences (10 transcripts were omitted due to their short length and/or their lack of predicted M1-M3 domains), one from *Musca domestica* [GenBank accession number AFP89966.1], 59 IR and 14 iGluR sequences identified in *D. melanogaster* [38], and 44 IR sequences from *Anopheles gambiae* [124]. For construction of the OBP dendogram, all 29 putative *C. stygia* sequences were analysed with 52 from *D. melanogaster* [125]. The OR and GR data set contained 43 and eight amino acid sequences, respectively, from *C. stygia* (seven OR and 13 GR transcripts were omitted due to their short length and/or lack of overlap when aligned) and 60 and 5, respectively, from *D. melanogaster*. Blowfly OR and GR sequences available from the NCBI database were also included. For the SNMP dendogram, all three putative *C. stygia* sequences were analysed with two SNMPs from *D. melanogaster* and an additional two SNMPs from *A. gambiae*. The esterase and Cytochrome P450 data sets contained 29 and 70 *C. stygia* sequences, respectively (12 esterase transcripts and 27 Cytochrome P450 transcripts were omitted due to their short length) and 35 and 85 sequences, respectively, from *D. melanogaster*. Protein names and GenBank accession numbers of genes used for building phylogenetic trees are listed in Additional file 4.

## Availability of supporting data

All supporting data is included within the article and its additional files. Candidate chemosensory genes were submitted to the National Center for Biotechnology Information (NCBI) and can be access at http://www.ncbi.nlm.nih.gov/gquery/?term=Calliphora+stygia [GenBank: KJ702047-KJ702310]. Phylogenetic trees and the underlying alignments are available at the CSIRO Data Portal (http://dx.doi.org/10.4225/08/54FF7B03DB181).

## Additional files

Additional file 1:
**Results of Blast against NCBI non-redundant protein database using predicted** ***C. stygia*** **transcripts as queries.** List of transcript ID, transcript length, and best BLASTp hit. Additional file 2:
**Candidate** ***C. stygia*** **antennal chemosensory genes.** Tables outline gene name, length, best BLASTx hit, and predicted domains. Additional file 3:
**Antennal expression levels of candidate** ***C. stygia*** **odorant receptors.**
Additional file 4:
**GenBank accession numbers of chemosensory genes, from organisms other than** ***C. stygia,*** **used in phylogenetic analyses.**

## Abbreviations

CSP: Chemosensory protein
GO: Gene Ontology
GR: Gustatory receptor
iGluR: Ionotropic glutamate receptor
IR: Ionotropic receptor
OBP: Odorant binding protein
OR: Odorant receptor
Orco: Odorant receptor co-receptor
ORN: Olfactory receptor neuron
SNMP: Sensory neuron membrane protein

## References

[1] Kurtovic A, Widmer A, Dickson B (2007). A single class of olfactory neurons mediates behavioural responses to a *Drosophila* sex pheromone. Nature.

[2] Becher P, LFlick G, Rozpedowska E, Schmidt A, Hagman A, Lebreton S (2012). Yeast, not fruit volatiles mediate *Drosophila melanogaster* attraction, oviposition and development. Funct Ecol.

[3] Birkett MA, Agelopoulos N, Jensen K-MV, Jespersen JB, Pickett JA, Prijs HJ (2004). The role of volatile semiochemicals in mediating host location and selection by nuisance and disease-transmitting cattle flies. Med Vet Entomol.

[4] Stensmyr M, Dweck H, Farhan A, Ibba I, Strutz A, Mukunda L (2012). A conserved dedicated olfactory circuit for detecting harmful microbes in *Drosophila*. Cell.

[5] Ashworth J, Wall R (1994). Responses of the sheep blowflies *Lucilia sericata* and *L. cuprina* to odour and the development of semiochemical baits. Med Vet Entomol.

[6] Byrd J, Castner J, Byrd J, Castner J (2009). Insects of forensic importance. Forensic entomology: the utility of arthropods in legal investigations.

[7] LeBlanc H, Logan J, Amendt J, Goff M, Campobasso C, Grassberger M (2010). Exploiting insect olfaction in forensic entomology. Current concepts in forensic entomology.

[8] Amendt J, Goff M, Campobasso C, Grassberger M (2010). Current concepts in forensic entomology.

[9] Aak A, Birkemoe T, Mehl R (2010). Blowfly (Diptera: Calliphoridae) damage on stockfish in northern Norway: pest species, damage assessment, and the potential of mass trapping. J Pest Sci.

[10] Fraser A, Broom D (1990). Farm animal behaviour and welfare.

[11] Green A (1951). The control of blowflies infesting slaughter-houses I: field observations of the habits of blowflies. Ann Appl Biol.

[12] Paul A, Ahmad N, Lee H, Ariff A, Saranum M, Maicker A (2009). Maggot debridement therapy with Lucilia cuprina: a comparison with conventional debridement in diabetic foot ulcers. Int Wound J.

[13] Tantawi T, Williams K, Villet M (2010). An accidental but safe and effective use of Lucilia cuprina (Diptera: Calliphoidae) in maggot debridement therapy in Alexandria, Egypt. J Med Entomol.

[14] Dekeirsschieter J, Verheggen FJ, Gahy M, Hubrecht F, Bourguignon L, Lognay G (2009). Cadaveric volatile organic compounds released by decaying pig carcasses (*Sus domesticus* L.) in different biotopes. Forensic Sci Int.

[15] Hädrich C, Ortmann C, Reisch R, Liebing G, Ahlers H, Mall G (2010). An electronic body-tracking dog?. Int J Legal Med.

[16] Paczkowski S, Maibaum F, Paczkowska M, Schütz S (2012). Decaying mouse volatiles percieved by Calliphora vicina Rob.-Desc. J Forensic Sci.

[17] Statheropoulos M, Spiliopoulou C, Agapiou A (2005). A study of volatile organic compounds evolved from the decaying human body. Forensic Sci Int.

[18] Vass AA, Barshick S-A, Sega G, Caton J, Skeen JT, Love JC (2002). Decomposition chemistry of human remains: a new methodology for determining the postmortem interval. J Forensic Sci.

[19] Vass AA, Smith RR, Thompson CV, Burnett MN, Dulgerian N, Eckenrode BA (2008). Odor analysis of decomposing buried human remains. J Forensic Sci.

[20] Vass AA, Smith RR, Thompson CV, Burnett MN, Wolf DA, Synstelien JA (2004). Decompositional odor analysis database. J Forensic Sci.

[21] Anderson GS, Byrd JH, Castner JL (2001). Insect succession on carrion and its relationship to determining time of death. Forensic entomology: the utility of arthropods in legal investigations.

[22] Aak A, Knudsen G (2011). Sex differences in olfaction-mediated visual acuity in blowflies and its consequences for gender-specific trapping. Entomol Exp et Applicata.

[23] Frederickx CE, Dekeirsschieter J, Verheggen FJ, Haubruge E (2012). Responses of *Lucilia sericata* Meigen (Diptera: Calliphoridae) to cadaveric volatile organic compounds. J Forensic Sci.

[24] Huotari M, Mela M (1996). Blowfly olfactory biosensor’s sensitivity and specificity. Sensors Actuators B.

[25] Kelling F, Biancaniello G, den Otter C (2002). Electrophysiological characterization of olfactory cell types in the antennae and palps of the housefly. J Insect Physiol.

[26] Wasserman S, Itagaki H (2003). The olfactory responses of the antenna and maxillary palp of the fleshfly, *Neobelliera bullata* (Diptera: Sarcophagidae), and their sensitivity to blockage of nitric oxide synthase. J Insect Physiol.

[27] ASTM Standard E1618 (2011). “Standard test method for ignitable liquid residues in extracts from fire debris samples by gas chromatography-mass spectrometry”.

[28] Hoffman EM, Curran AM, Dulgerian N, Stockham RA, Eckenrode BA (2009). Characterisation of the volatile organic compounds present in the headspace of decomposing human remains. Forensic Sci Int.

[29] Statheropoulos M, Agapiou A, Zorba E, Mikedi K, Karma S, Pallis GC (2011). Combined chemical and optical methods for monitoring the early decay stages of surrogate human models. Forensic Sci Int.

[30] Stensmyr M, Urru I, Collu I, Celander M, Hansson BS, Angioy A-M (2002). Rotting smell of dead-horse arum florets. Nature.

[31] Cossé AA, Baker TC (1996). House flies and pig manure volatiles: wind tunnel behavioural studies and electrophysiological evaluations. J Agri Entomol.

[32] Park KC, Cork A (1999). Electrophysiological responses of antennal receptor neurons in female Australian sheep blowflies, *Lucilia cuprina*, to host odours. J Insect Physiol.

[33] Zhu J, Chaudhury MF, Tangtrakulwanich K, Skoda SR (2013). Identification of oviposition attractants of the secondary screwworm, *Cochliomyia macellaria* (F.) released from rotten chicken liver. J Chem Ecol.

[34] Hallem E, Ho M, Carlson JR (2004). The molecular basis of odor coding in the *Drosophila* antenna. Cell.

[35] Clyne PJ, Warr CG, Freeman M, Lessing D, Kim JW, Carlson JR (1999). A novel family of divergent seven-transmembrane proteins: candidate odorant receptors in *Drosophila*. Neuron.

[36] Jones WD, Cayirlioglu P, Kadow IG, Vosshall LB (2007). Two chemosensory receptors together mediate carbon dioxide detecion in *Drosophila*. Nature.

[37] Vosshall LB, Stocker R (2007). Molecular architecture of smell and taste in *Drosophila*. Annu Rev Neurosci.

[38] Benton R, Vannice KS, Gomez-Diaz C, Vosshall LB (2009). Variant ionotropic glutamate receptors as chemosensory receptors in *Drosophila*. Cell.

[39] Benton R, Vannice K, Vosshall LB (2007). An essential role for a CD36-related receptor in pheromone detection in Drosophila. Nature.

[40] Jin X, Ha T, Smith D (2008). SNMP is a signaling component required for pheromone sensitivity in *Drosophila*. Proc Natl Acad Sci.

[41] Kaissling K (2001). Olfactory perireceptor and receptor events in moths: a kinetic model. Chem Senses.

[42] Leal W, Chen A, Ishida Y, Chiang V, Erickson M, Morgan T (2005). Kinetics and molecular properties of pheromone binding and release. Proc Natl Acad Sci.

[43] Bohbot J, Sobrio F, Lucas P, Magnan-Le Meillour P (1998). Functional characterization of a new class of odorant-binding proteins in the moth Mamestra brassicae. Biochem Biophys Res Commun.

[44] Pelosi P, Zhou J, Ban L, Calvello M (2006). Soluble proteins in insect chemical communication. Cell Mol Life Sci.

[45] Vogt R, Blomquist G, Vogt R (2003). Biochemical diversity of odor detection: OBPs, ODEs and SNMPs. Insect pheromone biochemistry and molecular biology.

[46] Martin F, Boto T, Gomez-Diaz C, Alcorta E (2013). Elements of olfactory reception in adult *Drosophila melanogaster*. Anotomical Record.

[47] Oakeshott J, Claudianos C, Campbell P, Newcomb RD, Russell R, Gillbert L, Iatrou K, Gill S (2005). Biochemical genetic and genomics of insect esterases. Comprehensive Molecular Insect Science.

[48] Fox A, Pitts R, Robertson H, Carlson JR, Zwiebel L (2001). Candidate odor receptors from the malaria vector mosquito, *Anopheles gambiae*. Proc Natl Acad Sci.

[49] Zhou X, Slone J, Rokas A, Berger S, Leibig J, Ray A (2012). Phylogenetic and transcriptomic analysis of chemosensory receptors in a pair of divergent ant species reveals sex-specific signatures of odor coding. PLoS Genet.

[50] Andersson MN, Grosse-Wilde E, Keeling CI, Bengtsson JM, Yuen MMS, Li M (2013). Antennal transcriptome analysis of the chemosensory gene families in the tree kiling dark beetles, *Ips typographus* and *Dendroctonus ponderosae* (Coleoptera: Curculionidae: Scolytinae). BMC Genomics.

[51] Bengtsson JM, Trona F, Montagné N, Anfora G, Ignall R, Witzgall P (2012). Putative chemosensory receptors of the codling moth, *Cydia pomonella*, identified by antennal transcriptome analysis. PLoS One.

[52] Glaser N, Gallot A, Legeai F, Montagné N, Poivet E, Harry M (2013). Candidate chemosensory genes in the stemborer *Sesamia nonagrioides*. Int J Biol Sci.

[53] Legeai F, Malpel S, Montagné N, Monsempes C, Cousserans F, Merlin C (2011). An expressed sequence tag collection from the male antennae of the Noctuid moth *Spodoptera littoralis*: a resource for olfactory and pheromone detection research. BMC Genomics.

[54] Vera J, Wheat C, Fescemyer H, Frilander M, Crawford D, Hanski I (2008). Rapid transcriptome characterization for a nonmodel organism using 454 pyrosequencing. Mol Ecol.

[55] Vogel H, Heidel A, Heckel D, Groot A (2010). Transcriptome analysis of the sex pheromone gland of the noctuid moth *Heliothis virescens*. BMC Genomics.

[56] Wang K-W, Luan J-B, Li J-M, Bao Y-Y, Zhang C-X, Lui S-S (2010). *De novo* characterization of a whitefly transcriptome and analysis of its gene expression during development. BMC Genomics.

[57] Lee S, Chen Z, McGrath A, Good R, Batterham P (2011). Identification, analysis, and linkage mapping of expressed sequence tags from the Australian sheep blowfly. BMC Genomics.

[58] Wang X, Zhong M, Wen J, Cai J, Jiang H, Liu Y (2011). Molecular characterization and expression pattern of an odorant receptor from the myiasis-causing blowfly, *Lucilia sericata* (Diptera: Calliphoridae). Parasitol Res.

[59] NCBI. National Center for Biotechnology Information (2012). U.S. National Library of Medicine.

[60] Krieger J, Klink O, Mohl C, Raming K, Breer H (2003). A candidate olfactory receptor subtype highly conserved across different insect orders. J Comp Physiol A Neuroethol Sens Neural Behav Physiol.

[61] Olafson P (2013). Molecular characterization and immunolocalization of the olfactory co-receptor Orco from two blood-feeding muscid flies, the stable fly (*Stomoxys calcitrans*, L.) and the horn fly (*Haematobia irritans irritans*, L.). Insect Mol Biol.

[62] Olafson P, Lohmeyer K, Dowd S (2010). Analysis of expressed sequence tags from a significant livestock pest, the stable fly (*Stromoxys calcitrans*), identifies transcripts with a putative role in chemosensation and sex determination. Arch Insect Biochem Physiol.

[63] Wang X, Zhong M, Liu Q, Sanaa MA, Wu C, Wen J (2013). Molecular characterization of the carbon dioxide receptor in the oriental latrine fly, *Chrysomya megacephala* (Dipters: Calliphoridae). Parasitol Res.

[64] Norris KR, Keast A, Crocker RL, Christian CS (1959). The ecology of sheep blowflies in Australia. Biogeography and Ecology in Australia.

[65] George KA, Archer M, Green LM, Conlan XA, Toop T (2009). Effect of morphine on the growth rate of *Calliphora stygia* (Fabricius) (Diptera: Calliphoridae) and possible implications for forensic entomology. Forensic Sci Int.

[66] Hulbert AJ, Usher MJ, Wallman JF (2004). Food consumption and individual lifespan of adults of the blowfly, *Calliphora stygia*: a test of the ‘rate of living’ theory of aging. Exp Gerontol.

[67] Parry S, Linton SM, Francis PS, O’Donnell MJ MJ, Toop T (2011). Accumulation and excretion of morphine by *Calliphora stygia*, and Australian blow fly species of forensic importance. J Insect Physiol.

[68] Ujvari B, Wallman JF, Madsen T, Whelan M, Hulbert AJ (2009). Experimental studies of blowfly (*Calliphora stygia*) longevity: a little dietary fat is beneficial but too much is detrimental. Comp Biochem Physiol A.

[69] Clyne PJ, Warr CG, Carlson JR (2000). Candidate taste receptors in *Drosophila*. Science.

[70] Kwon J, Dahanukar A, Weiss LA, Carlson JR (2007). The molecular basis of CO2 reception in *Drosophila*. Proc Natl Acad Sci.

[71] Ni L, Bronk P, Chang EC, Lowell AM, Flam JO, Panzano VC (2013). A gustatory receptor paralogue controls rapid warmth avoidance in *Drosophila*. Nat Lett.

[72] Slone J, Daniels J, Amrein H (2007). Sugar receptors in *Drosophila*. Curr Biol.

[73] Croset V, Rytz R, Cummins SF, Budd A, Brawand D, Kaessmann H (2010). Ancient protostome origin of chemosensory ionotropic glutamate receptors and the evolution of insect taste and olfaction. PLoS Genet.

[74] Shanbhag S, Müller B, Steinbrecht R (1999). Atlas of olfactory organs of *Drosophila melanogaster* 1. Types, external organization, innervation and distribution of olfactory sensilla. Int J Insect Morphol Embryol.

[75] Abuin L, Bargeton B, Ulbrich MH, Isacoff EY, Kellenberger S, Benton R (2011). Functional architecture of olfactory ionotropic glutamate receptors. Neuron.

[76] Hekmat-Scafe D, Scafe C, McKinney A, Tanouye M (2002). Genome-wide analysis of the odorant-binding protein gene familiy in *Drosophila melanogaster*. Genome Res.

[77] Sánchez-Gracia A, Vieira F, Rozas J (2009). Molecular evolution of the major chemosensory gene families in insects. Heredity.

[78] Jacquin-Joly E, Legeai F, Montagné N, Monsempes C, François M-C, Poulain J (2012). Candidate chemosensory genes in female antennae of the noctuid moth *Spodoptera littoralis*. Int J Biol Sci.

[79] Kim M-S, Repp A, Smith DP (1998). LUSH odorant-binding protein mediates chemosensory responses to alcohols in *Drosophila melanogaster*. Genetics Soc Am.

[80] Xu P, Zwiebel L, Smith DP (2003). Identification of a distinct family of genes encoding atypical odorant-binding proteins in the malaria vector mosquito, *Anopheles gambiae*. Insect Mol Biol.

[81] Feyereisen R (2006). Evolution of insect P450. Biochem Soc Trans.

[82] Feyereisen R, Gilbert LI (2012). Insect CYP genes and P450 enzymes. Insect molecular biology and biochemistry.

[83] Jackson CJ, Lui J-W, Carr PD, Younus F, Coppin C, Meirelles T, Lethier M, Pandey G, Ollis DL, Russell RJ, Weik M, Oakeshott JG (2013). Structure and function of an insect α-carboxylesterase (αEsterase7) associated with insecticide resistnace. Proc Natl Acad Sci.

[84] Newcomb RD (1997). A single amino acid substitution converts a carboxylesterase to an organophosphorous hydrolase and confers insecticide resistance on a blowfly. Proc Natl Acad Sci.

[85] Daborn P, Boundy S, Yen J, Pittendrigh B, Ffrench-Constant R (2001). DDT resistance in *Drosophila* correlates with *Cyp6g1* over-expression and confers cross-resistance to the neonicotinoid imidacloprid. Mol Gen Genomics.

[86] Grosse-Wilde E, Kuebler LS, Bucks S, Vogel H, Wicher D, Hansson BS (2012). Antennal transcriptome of *Manduca sexta*. Proc Natl Acad Sci.

[87] Zhang M, Yu H, Yang Y, Song C, Hu X, Zhang G (2013). Analysis of the transcriptome of blowfly *Chrysomya megacephala* (Fabricius) larvae in response to different edible oils. PLoS One.

[88] Liu Y, Gu S, Zhang Y, Guo Y, Wang G (2012). Candidate olfaction genes identified within the *Helicoverpa armigera* antennal transcriptome. PLoS One.

[89] Mitchell R, Hughes D, Leutje C, Millar J, Soriano-Agatón F, Hanks L (2012). Sequencing and characterizing odorant receptors of the cerambycid beetle *Megacyllene caryae*. Insect Biochem Mol Biol.

[90] Pitts RJ, Rinker DC, Jones PL, Rokas A, Zwiebel LJ (2011). Transcriptome profiling of chemosensory appendages in the malaria vector *Anopheles gambiae* reveals tissue- and sex-specific signatures of odor coding. BMC Genomics.

[91] Riveron J, Boto T, Alcorta E (2013). Transcriptional basis of the acclimation to high environmental temperature at the olfactory receptor organs of *Drosophila melanogaster*. BMC Genomics.

[92] Poivet E, Gallot A, Montagné N, Glaser N, Legeai F, Jacquin-Joly E (2013). A comparison of the olfactory gene repertoirs of adult and larvae in the noctuid moth *Spodoptera littoralis*. PLoS One.

[93] Rytz R, Croset V, Benton R (2013). Ionotropic receptors (IRs): chemosensory ionotropic glutamate receptors in *Drosophila* and beyond. Insect Biochem Mol Biol.

[94] Briscoe A, Macias-Muñoz A, Kozak K, Walters J, Yaun F, Jamie G (2013). Female behaviour drives expression and evolution of gustatory receptors in butterflies. PLoS Genet.

[95] Benton R (2008). Chemical sensing in *Drosophila*. Curr Opin Neurobiol.

[96] Montell C (2013). Gustatory receptors: not just for good taste. Curr Biol.

[97] Yao CA, Ingnell R, Carlson JR (2005). Chemosensory coding by neurons in the coeloconic sensilla of the *Drosophila* antenna. J Neurosci.

[98] Cooke M, Leeves N, White C (2003). Time profile of putrescine, cadaverine, indole and skatole in human saliva. Arch Oral Biol.

[99] Andersson MN, Videvall E, Walden K, Harris MO, Robertson H, Löfstedt C (2014). Sex- and tissue-specific profiles of chemosensory gene expression in herbivorous gall-inducing fly (Diptera: Cecidomyiidae). BMC Genomics.

[100] Rinker DC, Zhou X, Pitts RJ, Consortium TA, Rokas A, Zwiebel LJ (2013). Antennal transcriptome profiles of anopheline mosquitoes reveal human host olfactory specialization in *Anopheles gambiae*. BMC Genomics.

[101] Dekeirsschieter J, Frederick CE, Lognay G, Brostaux Y, Berheggen FJ, Haubruge E (2013). Electrophysiological and behavioral responses of *Thanatophilus sinuatus* Fabricius (Coleoptera: Silphidae) to selected cadaveric volatile organic compounds. J Forensic Sci.

[102] Aak A, Knudsen GK, Soleng A (2010). Wind tunnel behavioural response and field trapping of the blowfly *Calliphora vicina*. Med Vet Entomol.

[103] Archer M, Elgar MA (2003). Female breeding-site preferences and larval feeding strategies of carrion-breeding Calliphoridae and Sarcophagidae (Diptera): a quantitative analysis. Aust J Zool.

[104] Wall R, Green CH, French N, Morgan KL (1992). Development of an attractive target for the sheep blow fly *Lucilia sericata*. Med Vet Entomol.

[105] Ejima A, Smith B, Lucas C, van der Goes van Naters W, Miller C, Carlson J (2007). Generalization of courtship learning in *Drosophila* in mediated by *cis-*vaccenyl acetate. Curr Biol.

[106] Uebel EC, Sonnet PE, Bierl BA, Miller RW (1975). Sex pheromone of the stable fly: isolation and preliminary identification of compounds that induce mating strike behavior. J Chem Ecol.

[107] Uebel EC, Sonnet PE, Miller RW, Beroza M (1975). Sex pheromone of the face fly, *Musca autumnalis* De Geer (Diptera: Muscidae). J Chem Ecol.

[108] Carlson DA, Mayer MS, Silhacek DL, James JD, Beroza M (1971). Sex attractant pheromone of the house fly: isolation, identifcation and synthesis. Science.

[109] George K, Archer M, Toop T (2012). Effects of bait age, larval chemical cues and nutrient depletion on colonization by forensically important callihorid and sarcophagid flies. Med Vet Entomol.

[110] Grabherr M, Haas B, Yassour M, Levin J, Thompson D, Amit I (2011). Full-length transcriptome assembly from RNA-Seq data without a reference genome. Nat Biotechnol.

[111] Haas B, Papanicolaou A, Yassour M, Grabherr M, Blood P, Bowden J (2013). *De novo* transcript sequence reconstruction from RNA-seq using the Trinity platform for reference generation and analysis. Nat Protoc.

[112] DEW (2014). Digital expression on the web.

[113] Langmead B, Salzberg L (2012). Fast gapped-read alignment with Bowtie 2. Nat Methods.

[114] Forster SC, Finkel AM, Gould JA, Hertzog PJ (2013). RNA-eXpress annotated novel transcript features in RNA-seq data. Bioinformatics.

[115] Conesa A, Götz S, García-Gómez J, Terol J, Talón M, Robles M (2005). Blast2GO: a universal tool for annotation, visualization and analysis in functional genomics research. Bioinformatics.

[116] Götz S, García-Gómez J, Terol J, Williams T, Nagaraj S, Nueda M (2008). High-throughput functional annotation and data mining with the Blast2GO suite. Nucleic Acids Res.

[117] Quevillon E, Silventoinen V, Pillai S, Harte N, Mulder N, Apweiler R (2005). InterProScan: protein domains identifier. Nucleic Acids Res.

[118] Käll L, Krogh A, Sonnhammer E (2004). A combined transmembrane topology and signal peptide prediction method. J Mol Biol.

[119] Gou S, Kim JW (2007). Molecular evolution of *Drosophila* odorant receptor genes. Mol Biol Evol.

[120] Katoh K, Misawa K, Kuma K-I, Miyata T (2002). MAFFT: a novel method for rapid multiple sequence alignment based on fast Fourier transform. Nucleic Acids Res.

[121] Tamura K, Peterson D, Peterson N, Stecher G, Nei M, Kumar S (2011). MEGA5: molecular evolution genetics analysis using maximum liklihood evolutionary distance, and maximum parsimony methods. Mol Biol Evol.

[122] Rambaut A (2007). FigTree, v1.4.

[123] Inkscape. v.0.48.4. 2013. http://inkscape.org.

[124] Megy K, Emrich SJ, Lawson D, Campbell D, Dialynas E, Hughes DST, et al. VectorBase: improvements to a bioinformatics resource for invertebrate vector genomics. Nucleic Acids Res. 2012; 40(D1): p. D729-D34. doi:10.1093/nar/gkr1089. 10.1093/nar/gkr1089 PMC3245112 22135296

[125] Marygold S, Leyland P, Seal R, Goodman J, Thurmond J, Strelets V (2013). FlyBase improvements to the bibliography. Nucleic Acids Res.

